# “Oriental anthropometry” in plastic surgery

**DOI:** 10.4103/0970-0358.44947

**Published:** 2008

**Authors:** Vasco Senna-Fernandes

**Affiliations:** Department of Aesthetic Therapies Research, Academia Brasileira de Arte e Ciência Oriental, Sohaku-In Foundation, Rio de Janeiro, Brazil

**Keywords:** Acupoint and aesthetic-locus, oriental-anthropometry, plastic-surgery

## Abstract

**Background:**

According to Chinese medicine, the acupuncture-points' (acupoints) locations are proportionally and symmetrically distributed in well-defined compartment zones on the human body surface Oriental Anthropometry” (OA). Acupoints, if considered as aesthetic-loci, might be useful as reference guides in plastic surgery (PS).

**Aim:**

This study aimed to use aesthetic-loci as anatomical reference in surgical marking of Aesthetic Plastic Surgery.

**Method:**

This was an observational study based on aesthetic surgeries performed in private clinic. This study was based on 106 cases, comprising of 102 women and 4 men, with ages varying from 07 to 73 years, and with heights of between 1.34 m and 1.80 m. Patients were submitted to aesthetic surgical planning by relating aesthetic-loci to conventional surgical marking, including breast surgeries, abdominoplasty, rhytidoplasty, blepharoplasty, and hair implant. The aesthetic-surgical-outcome (ASO) of the patients was assessed by a team of plastic surgeons (who were not involved in the surgical procedures) over a follow-up period of one year by using a numeric-rating-scale in percentage (%) terms. A four-point-verbal-rating-scale was used to record the patients' opinion of therapeutic-satisfaction (TS).

**Results:**

ASO was 75.3 ± 9.4% and TS indicated that most patients (58.5%) obtained “good” results. Of the remainder, 38.7% found the results “excellent”, and 2.8% found them “fair”.

**Discussion and Conclusion:**

The data suggested that the use of aesthetic-loci may be a useful tool for PS as an anatomical reference for surgical marking. However, further investigation is required to assess the efficacy of the OA by providing the patients more reliable balance and harmony in facial and body contours surgeries.

## INTRODUCTION

Anthropometry is defined as the science of measuring the human body and its parts.[1] During the Renaissance, anthropometry was associated with the fine arts and sculpture. It was the most precise and ideal form for expressing the heroic dimensions of the human body. The artist Leonardo da Vinci was widely recognized for his interest and focus on human anatomy, and his use of the system of idealized measurements when working on the human figure.[2]

In spite of its history of evolution up until the end of the 20^th^ century, Plastic and Reconstructive Surgery has used Renaissance anthropometry as the source in determining bodily proportions. This was mainly in facial cosmetic and body contour procedures.[3]

While in the Western World, anthropometry was used initially for artistic purposes and later in scientific studies, in the Far East it was standardized essentially in connection with “energy therapy”.[4–6] Since then, the System of Meridians and Acupuncture Points (SMAP) has been considered as anthropometry because it consists of well-defined compartments.

In the Far East, the “Traditional Chinese Medicine's (TCM) Anthropometry” dates back more than 2990 years. An early description occurs in - “*Lingshu - Huangdi Nei Ching” (The Miraculous Pivot - The Yellow Emperor's Classic of Internal)*, which was considered to be the mother book of the Chinese traditional acupuncture and one of the oldest medical compilation book in China, written by numerous medical authors during the “Warring States Period” (475-221 BC).[6,7] It was used in the precise location of the meridians and acupuncture points (acupoints) for therapeutic purposes. From the Song Dynasty (960-1279 AD), the first bronze statues were idealized with topographical mapping of the meridians and their respective acupoints by *Wang Wei-Yi* (approx. 987–1067 AD), in 1026, and this was later improved in the Ming Dynasty (1368-1644), as the study of acupuncture gradually became more sophisticated.[8–10] According to the last revised edition of “Standard Acupuncture Nomenclature” by the WHO Scientific Group to Adopt a Standard International Acupuncture Nomenclature (Geneva, 1989), and the WHO Regional Office for the Western Pacific (Manila, 1991), the essence of the SMAP proportionality and symmetry is still preserved in spite of having suffered constant changes during the course of Chinese history.[6,11,12]

In acupuncture practice, the exact location of the acupoints has always been a theme of controversial discussion.[13] For greater precision in the location of the acupoints several methods of measurement were developed, such as the *(a)* Digital Measurements (thumb, middle finger, two-fingers, two-fingers and half, index-finger and, four-finger measurements) - DM, *(b)* Eye Measurement - EM, *(c)* Proportional Measurement - PM, and *(d)* Anatomical Landmark Measurements (superficial and convenient measurements) - ALM.[6,14–18] According to previous studies, the human body is divided into very well defined sections using a pattern of units, the *cun* (Chinese anatomical inch), which is used to determine the location of acupoints from a set of systematic measurements based on the proportionality and symmetry of the body compartments.[6]

**Figure 1:**
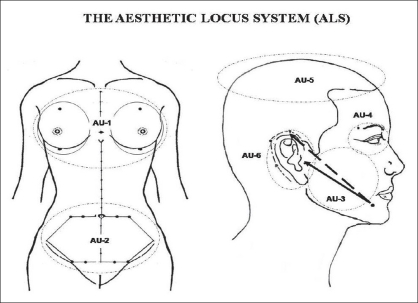
ALS is consisted of 24 aesthetic loci that play important role on 6 aesthetic units (AU)

Therefore, the locus of some acupoints can be used as an anatomical reference guide for surgical demarcation in plastic surgery. It consists of a group of body surface sites, called the “aesthetic-loci”, which coincide with the acupoints or lie close to the same, and which is applied as an anatomical reference to the aesthetic units (AU) during surgical planning in Plastic Surgery[19–21] [Figure 1, Table 1].

**Table 1:** Aesthetic Loci System: Classification and Nomenclature

*Aesthetic Unit (AU)*	*Aesthetic Locus (AL)*	

	*Standard Nomenclature*	*Simplified Nomenclature*
AU_1_: Breast Region	AL-CV.17 (Tanzhong)	M_1_
	AL-ST.17 (Ruzhong)	M_2_
	AL-ST.15 (Wuyi)	M_3_
	AL-ST.18 (Rugen)	M_4_
	AL-LR.14 (Qimen)	M_5_
AU_2_: Abdominal Region	AL-SP.15 (Daheng)	Ab_1_
	AL-SP.12 (Chongmen)	Ab_2_
	AL-ST.25 (Tianshu)	Ab_3_
	AL-ST.30 (Qichong)	Ab_4_
	AL-CV.8 (Shenque)	Ab_5_
AU_3_: Facial Region	AL-Ex.XF.6 (Jiangchengjian)	F_1_
	AL-SI.19 (Tinggong)	F_2_
	AL-TE.20 (Jiasun)	F_3_
AU_4_: Periorbitary Region	AL-TE.23 (Sizhukong)	PO_1_
	AL-UB.l (Jingming)	PO_2_
AU_5_: Scalp Region	AL-HFLp	S_1_
AU_6_: Ear Region	AL-TE.23 (Sizhukong)	E_1_
	AL-TE.20 (Jiaosun)	E_2_
	AL-GB.1 (Tongziliao)	E_3_
	AL-TE.22 (Erheliao)	E_4_
	AL-ST.3 (Juliao)	E_5_
	AL-TE.17(Yifeng)	E_6_
	AL-TE.19 (Luxi)	E_7_
	AL-TE.18 (Chimai)	E_8_

AL - is a classification of locus (from acupoints) to aid the plastic surgeons' understanding; AU: Aesthetic Units; M: mammary locus; Ab: abdominal locus; F: facial locus; PO: periorbitary locus; S: Scalp locus, and E: ear locus; CV: conception vessel; GB: gall bladder meridian; LR: liver meridian; SP: spleen meridian; ST: stomach meridian; TE: Triple energizer meridian; UB: urinary meridian; and Ex.XF: face extra-point; HFLp: hair front line midpoint.

The Aesthetic Locus System (ALS) comprises of 24 sites of aesthetic-loci, being 2 single and 22 double in number, which are divided into 6 AU, being: (a) AU_1_ - breast region: 5 loci; (b) AU_2_ - abdomen region: 5 loci; (c) AU_3_ - face region: 3 loci; (d) AU_4_ - periorbital region: 2 loci; (e) AU_5_ - scalp region: 1 locus; (f) AU_6_ - ear region: 8 loci [Table 1].

The nomenclature of the aesthetic-loci is based on; (*i*) the standard nomenclature of ALS that consists of a standard alphabetic code, an alphanumeric code, and a Chinese phonetic alphabet of Han dialect (*Pinyin*). This nomenclature is suitable for those who already have acupuncture or TCM background. (*ii*) The simplified nomenclature of ALS consists of an alphanumeric code which is more suitable for those who do not have any experience in acupuncture, such as plastic surgeons. In this study, the aesthetic-loci are expressed in simplified nomenclature [Table 1].

## METHOD

**Figure 2:**
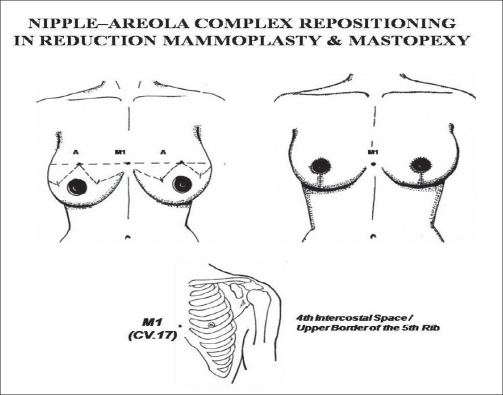
Nipple-Areolar Complex under the reference guide of M_1_ or CV.17 (Tanzhong).

**Figure 3:**
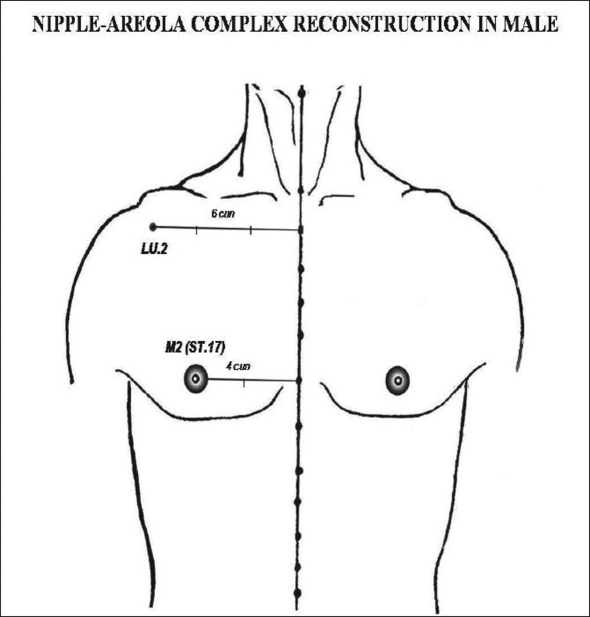
M_2_ or ST.17 (Rugen) is located 2/3 the distance between the acupoint LU.2 (Yunmen) and midline (6 cun)

**Figure 4:**
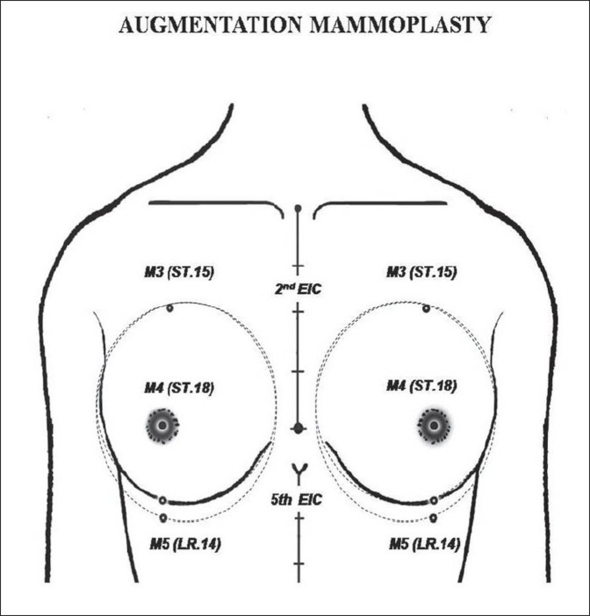
Demarcation of the breast implant pouch under the reference guide of mammary loci M_3_ or ST.15 (Wuyi), M_4_ or ST.18 (Rugen) and M_5_ or (Qimen).

This study was performed between 2005 and 2006 in the *Department of Aesthetic Therapies Research, Academia Brasileira de Arte e Ciência Oriental/ Colégio Brasileiro de Acupuntura* (ABACO/CBA), Rio de Janeiro, Brazil. The Ethical Committee of the ABACO/CBA, Rio de Janeiro/RJ, Brazil approved the research protocols, and all patients gave informed consent to this observational study. A hundred and six cases, consisting of 102 women and 4 men, with ages varying from 07 to 73 years, and measuring between 1.34 m and 1.80 m, were submitted to systematic surgical planning [Table 3]:

I) The patients were marked with aesthetic-loci as anatomical references according to each aesthetic unit [Table 1];

(a) In the breast region (AU_1_), the locus M_1_ (CV.17, *Tanzhong*) was used as to directly guide the nipple-areola complex center (NACc) repositioning, in mastopexy, and in reduction mammoplasty [Figure 2].[22,23] Since M_1_ is normally located at the anatomical and physiological level of the nipples in young female and nuliparas,[24] the NACc repositioning location was guided by this locus on the level of the 4^th^ intercostal space (upper border of the 5^th^ rib) or lower, but never above it. When M_1_ is associated to M_2_ (ST.17, *Ruzhong*) these loci were used to guide directly the nipple placement such as in Gynecomastia grade III[25] and Athelia corrections[26] [Figure 3]. Also in the AU_1_, the loci M_3_ (ST.15, *Wuyi*), M_4_ (ST.18, *Rugen*), and M_5_ (LR.14, *Qimen*) were used to determine the area of the breast implant placement (small or moderate size - 195-215 cc) to be undermined in augmentation mammoplasty[6,27] [Figure 4].

**Figure 5:**
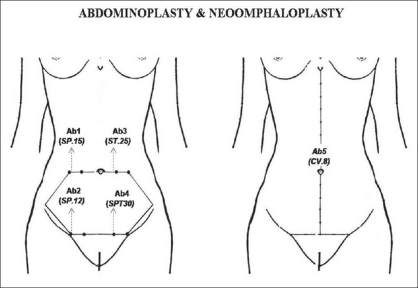
Abdominoplasty under the reference guide of abdominal points: (a) Ab_1_ or SP.15 (Daheng), Ab_2_ or SP.12 (Chongmen), Ab_3_ or ST.25 (Tianshu), and Ab_4_ or ST.30 (Qichong); are used as downward vector for traction, resection and fixation of the dermoadipose flap. (b) Ab_5_ or CV.8 (Shenquen) for umbilicus position marking during neoomphaloplasty.

In the abdominal region (AU_2_), the loci Ab_1_ (SP.15, *Daheng*) and Ab_2_ (SP.12, *Chongmen*), or Ab_3_ (ST.25, *Tianshu*) and Ab_4_ (ST.30, *Qichong*) were used to demarcate the abdominal area to be excised and to guide the downward traction of the flap and suture, in abdominoplasty[28,29] [Figure 5]. Also in the AU_2_, the locus Ab_5_ (CV.8, *Shenque*) was used to define the center of the new umbilicus site in the neo-omphaloplasty which is usually located 5 *cun* above the pubic region, being the pubic-xiphoid segment divided in 13 *cun*, as described in TCM [Figure 5].[6,29–31]

**Figure 6:**
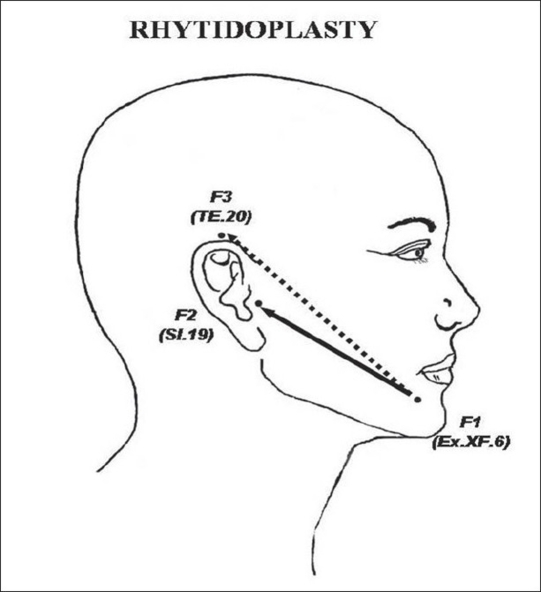
Rhytidoplasty guided by facial loci: F_1_ or Ex.XF.6 (Jiangchengjiang), F_2_ or SI.19 (Tinggong), and F_3_ or TE.20 (Jiaosun) during the facial flap traction.

In the facial region (AU_3_), the loci F_1_ (Ex.XF.6, *Jiangchengjian*), F_2_ (SI.19, *Tinggong*) and F_3_ (TE.20, *Jiasun*) were used to guide the cervicofacial traction direction in rhytidoplasty[29] [Figure 6].

**Figure 7:**
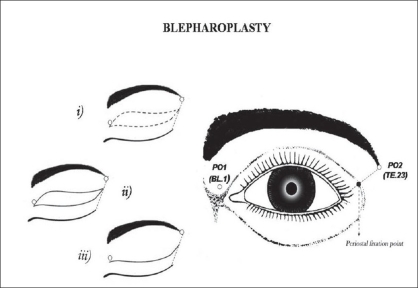
Periorbitary loci used in eyelid surgeries: (a) Upper blepharoplasty guided by PO_1_ or UB.1 (Jingming) and PO_2_ or TE.23 (Sizhukong) for marking of two extremities to avoid large upper eyelid scar. (b) Lateral canthopexy guided by PO_1_ for periostal fixation of the outer canthus ligament at the anterior and lateral aspects of the periorbitary wall.

In the periorbital region (AU_4_), the loci PO_1_ (TE.23, *Sizhukong*) and PO_2_ (UB.1,*Jingming*) were used to determine the extremities extension of the upper eyelid scar in blepharoplasty. Also in AU_4_, the locus PO_1_ (TE.23 *Sizhukong*) was used to guide the periostal fixation of the lateral canthus tendon in canthopexy[6,29,32] [Figure 7].

**Figure 8a:**
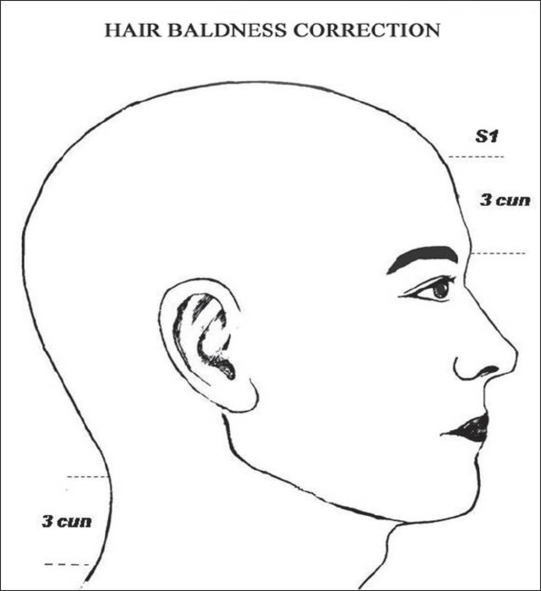
In Oriental Medicine the scalp loci (S_1_) is the hair front line midpoint of the scalp region, (3 cun above the glabellar region (Yintang point).

**Figure 8b:**
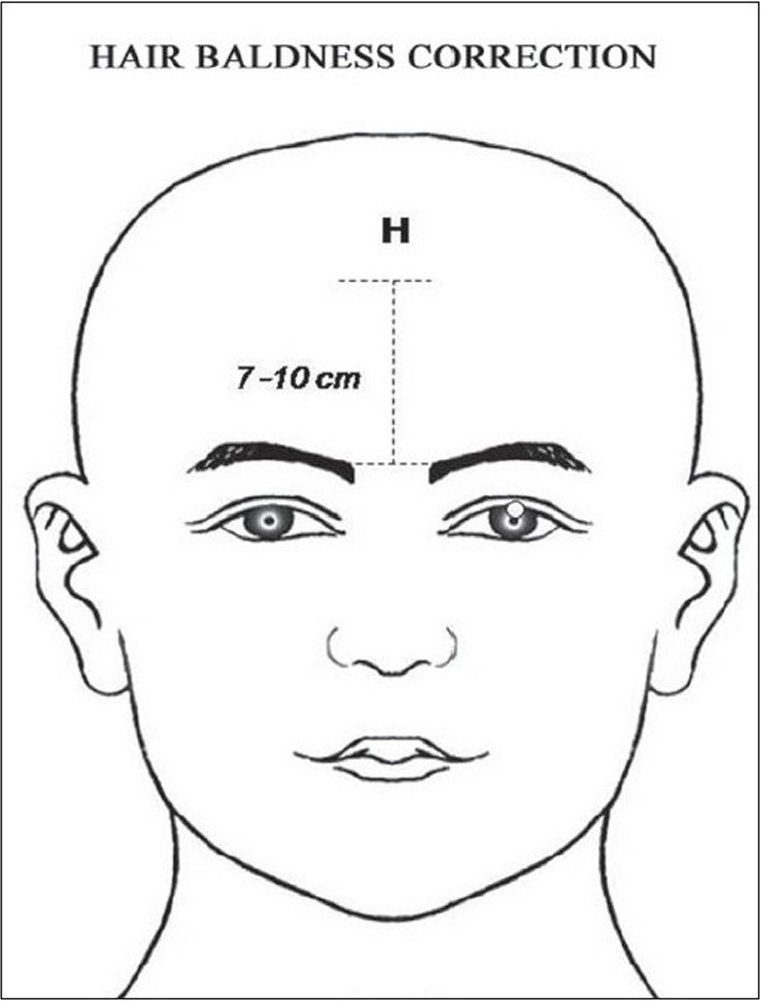
In Plastic Surgery, the “Point H” is located between 7 and 10 cm above the glabellar (or naso-frontal region).

In the scalp region (AU_5_), the locus S_1_ is located on the center of the forehead located at 3 *cun* above the gabelar region (*Yintang* point) which corresponds to the midpoint of the hair frontline (HFL) for baldness correction - hair implant procedure[6,33,34] [Figure 8].

**Figure 9:**
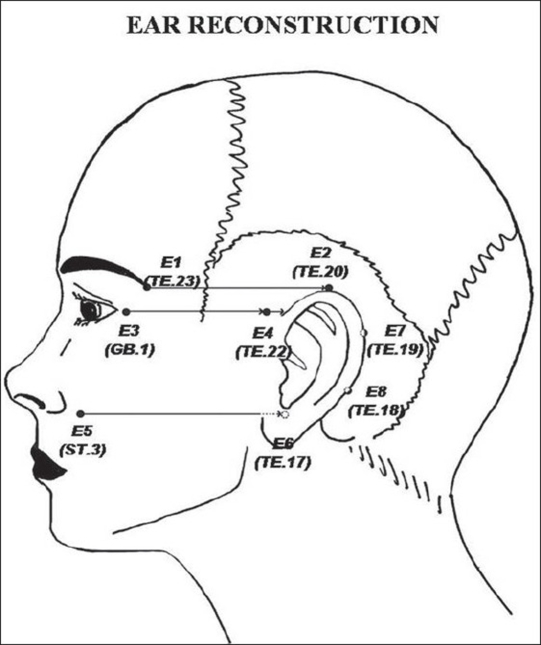
Ear loci guiding the anatomical location of the ear's framework: E_1_ (TE.23, Sizhukong), E_2_ (TE.20, Jiaosun), E_3_ (GB.1, Tongziliao), E_4_ (TE.22, ErHeliao), E_5_ (ST.3, Juliao), E_6_ (TE.17, Yifeng), E_7_ (TE.19 Luxi), and E_8_ (TE.18, Chimai).

In the ear region (AU_6_), the loci E_1_ (TE.23, *Sizhukong*), E_2_ (TE.20, *Jiaosun*), E_3_ (GB.1, *Tongziliao*), E_4_ (TE.22, *ErHeliao*), E_5_ (ST.3, *Juliao*), E_6_ (TE.17, *Yifeng*), E_7_ (TE.19 *Luxi*); E_8_ (TE.18, *Chimai*) were used to locate and design the ear's framework for partial or total ear reconstruction, such as in microtia and ear trauma[29] [Figure 9].

II) The conventional demarcation was carried out under the guidance of those aesthetic-loci in accordance with the need of each category of surgery.

III) The surgical interventions were performed according to the routine of each type of aesthetic procedure. During the surgical procedure, some aesthetic-loci were applied to guide the traction direction (facial and abdominal advancement flaps, as given examples).

IV) The aesthetic surgery outcome (ASO) of all patients was evaluated over a follow-up period of one year by a team composed by two experienced plastic surgeons who are board certified by the Brazilian Society of Plastic Surgery, one of them not being involved in the surgical procedures. ASO was based on subjective parameters of aesthetic results including *(a)* asymmetry, shape, and anatomical position of each aesthetic unit and *(b)* scar tension, quality, and deviation. Moreover, ASO was scored by a numeric rating scale in percentages (%) terms, “0” being the worst result and 100 the perfect result.[35] A four point verbal rating scale was used to record the patients' opinion of therapeutic satisfaction (TS) on the aesthetic results: 1-poor, 2-fair, 3-good, and 4-excellent.

## RESULTS

Patient female : male ratio was 25.5:1, with a mean age of 45.3 ± 12.5 years. Table 1 shows the classification and two types of nomenclatures of the aesthetic-loci. Table 2 refers to the standard anatomical location of each acupoint used as aesthetic-locus for this study.

**Table 2:** Location of Acupoints related to the Aesthetic Loci (AL)

*AL*	·	*Acupoint*	*Anatomical Location*	
M_1_	·	CV.17 (Tanzhong)	-	On the anterior midline of the sternum, at the level of the 4^th^ intercostal space, midway between the nipples, at the upper border of the 5^th^ rib.
M_2_	·	ST.17 (Ruzhong)	-	On the 4^th^ intercostal space, at the upper border of the 5^th^ rib, 4 cun laterally to CV.17, and so, at the center of the nipple.
M_3_	·	ST.15 (Wuyi)	-	On the chest, on the midclavicular line, in the 2^nd^ intercostal space, 4 cun lateral to the anterior midline.
M_4_	·	ST.18 (Rugen)	-	On the chest, on the midclavicular line, in the 5^th^ intercostal space, 4 cun lateral to the anterior midline.
M_5_	·	LR.14 (Qimen)	-	On the chest, on the midclavicular line, in the 6^th^ intercostal space, at the level of CV.14.
Ab_1_	·	SP.15 (Daheng)	-	On the abdomen, at the level of the umbilicus, 4 cun lateral to the anterior midline.
Ab_2_	·	SP.12 (Chongmen)	-	In the inguinal region, on the lateral side of the femoral artery, 3.5 cun lateral to the anterior midline.
Ab_3_	·	ST.25 (Tianshu)	-	On the abdomen, 2 cun lateral to the umbilicus.
Ab_4_	·	ST.30 (Qichong)	-	At the superior border of the pubic symphysis, 2 cun lateral to the anterior midline, at the level of CV.2.
Ab_5_		CV.8 (Shenque)	-	At the center of the umbilicus.
F_1_		Ex.XF.6 (Jiangchengjian)	-	1 cun lateral of the midline (CV.24)
F_2_	·	SI.19 (Tinggong)	-	Anterior to the tragus and posterior to the condyloid process of the mandible, in a depression formed when the mouth is opened.
F_3_	·	TE.20 (Jiaosun)	-	In the temporal region, within the hairline, superior to the apex of the ear.
PO_1_	·	TE.23 (Sizhukong)	-	In the depression at the lateral end of the eyebrow.
PO_2_	·	UB.l (Jingming)	-	On the face, 0.1 cun superior to the inner canthus when the eye is closed.
S_1_	·	HFLp	-	On the forehead, 3 cun above the glabelar region (Yintang Point)
E_1_	·	TE.23 (Sizhukong)	-	In the depression at the lateral end of the eyebrow.
E_2_	·	TE.20 (Jiaosun)	-	In the temporal region, within the hairline, superior to the apex of the ear.
E_3_		GB.1 (Tongziliao)	-	On the lateral face, 0.5 cun lateral to the outer canthus of the eye.
E_4_	·	TE.22 (Erheliao)	-	Anterior to the ear, on the hairline, level with the lateral canthus of the eye.
E5	·	ST.3 (Juliao)	-	On the face, level with the border of the ala nasi, in line with the pupil when the eyes are focused forward.
E_6_	·	TE.17 (Yifeng)	-	At the ear, in the depression between the mastoid process and the mandible, behind the ear lobe.
E_7_	·	TE.19 (Luxi)	-	On the mastoid bone, posterior to the ear, at the junction of the middle and upper third of the curve that connects TE.17 at the earlobe and TE.20 at the apex of the ear.
E_8_	·	TE.18 (Chimai)	-	On the mastoid bone, posterior to the ear, at the junction of the lower and middle third of the curve that connects TE.17 at the earlobe and TE.20 at the apex of the ear.

AL: Aesthehtic-locus; CV: conception vessel; GB: gall bladder meridian; LR: liver meridian; SP: spleen meridian; ST: stomach meridian; TE: Triple energizer meridian; UB: urinary meridian; and Ex.XF: face extra-point; HFLp: hair front line midpoint (3 cun above the midline of gabelar)

Table 3 shows the distribution of the relative frequency of each category of aesthetic surgery of this study, considering that breast reduction [Figure 10a–c] blepharoplasty [Figure 11a–c] and abdominoplasty were the most frequently used procedures.

Table 4 shows that on TS, most patients (58.5%) declared that the results were good, 38.7% reported excellent results and 2.8% fair results. No patient reported results in the poor category [Table 5]. Moreover, Table 4 also refers to the final results from ASO evaluated by the PS team over a follow-up period of one year with satisfactory outcomes. According to data, there were no severe complications recorded, such as scar asymmetry, tension or retraction, anatomical malposition or deviation of the AUs which could be directly related to inadequate surgical demarcation guided by aesthetic-loci (ASO: 75.3 ± 9.4%).

**Table 3:** Clinical series study using Aesthetic Loci in Plastic Surgery

*Surgical procedures*	*Aesthetic locus*	*Cases(n)*
· Reduction Mammoplasty	M_1_	35
· NAC Reconstruction	M_2_	2
· Augmentation Mammoplasty	M_3_, M_4_, M_5_	10
Abdominoplasty	Ab_1_, Ab_2_, Ab_3_, Ab_4_	23
Neoomphaloplasty	Ab_5_	(3)*
· Rhytidoplasty	F_1_, F_2_, F_3_	10
· Blepharoplasty	PO_1_, PO_2_	21
· Lateral Canthopexy	PO_1_	(10)**
· Hair Implant	S	3
· Ear Reconstruction	E_1_, E_2_, E_3_, E_4_, E_5_, E_6_, E_7_, E_8_	2

· Total		106

The distribution of the aesthetic-loci related to each type of surgical procedures and their relative frequencies (np/p).

* Within 23 abdominoplasties, three patients submitted to neoomphaloplasty.

** Within 21 blerophaplasty, ten patients underwent lateral canthopexy

**Table 4:** Surgical Procedures Evaluation

*Surgical Procedures*	*Therapeutic Satisfaction*					*ASO*
	
	*N*	*Poor*	*Fair*	*Good*	*Excellent*	*(NRS, %)*
Reduction Mammoplasty	35	-	-	27	8	76.8 ± 8.6
NAC Reconstruction	2	-	1	-	1	65.0 ± 2.0
Augmentation Mammoplasty	10	-	-	4	6	87.0 ± 5.5
Abdominoplasty	23	-	-	13	10	83.0 ± 5.2
Rhytidoplasty	10	-	-	4	6	84.7 ± 6.1
Blepharoplasty	21	-	-	11	10	80.2 ± 6.7
Hair Implant	3	-	1	2	-	78.3 ± 2.8
Ear Reconstruction	2	0	1	1	-	47.0 ± 4.5
Total	106	0	3	62	41	75.3 ± 9.4

		(0%)	(2.8%)	(58.5%)	(38.7%)	

The distribution of the patients' opinion of therapeutic satisfaction of each category of surgical procedure which consists of a four point verbal rating scale, being 1-poor, 2-fair, 3-good, 4-excellent. The distribution of the Aesthetic Surgery Outcome (ASO) was based on the plastic surgery team evaluation on a numeric ration scale (NRS) in percentage (%).

**Figure 10a:**
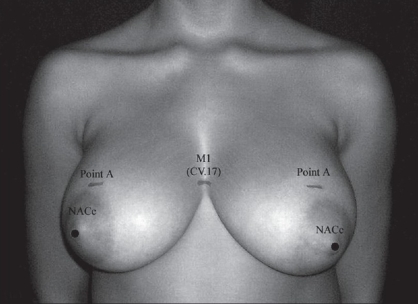
Case 1 is an example of a patient with moderate hypertrophic breasts who was submitted for reduction mammoplasty guided by the aesthetic locus M_1_(CV.17). Before surgery, M_1_ locus and Point A and NACc (nipple-areolar complex) were located.

**Figure 10b:**
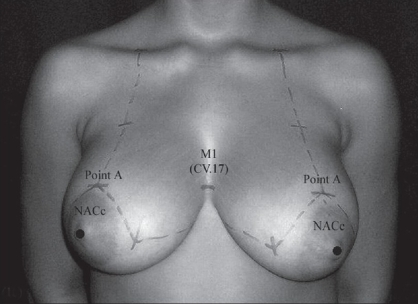
Conventional demarcation of reduction mammoplasty guided by the M_1_ locus

**Figure 10c:**
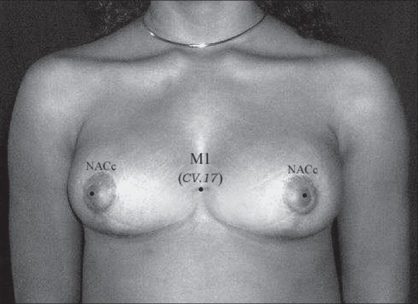
One year of follow-up. In this case, “Point A” and NACc (nippleareolar complex center) levels coincided to the M_1_ locus without any great changes of the NACc position.

**Figure 11a:**
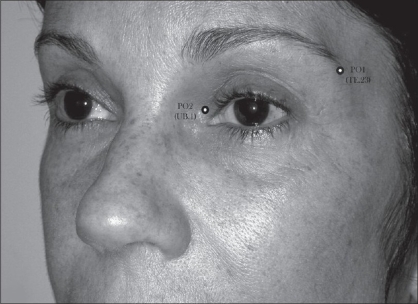
Patient underwent blepharoplasty with lateral canthopexy. (a) Before surgery, the upper eyelid correction was guided by the aesthetic loci, PO_1_ (TE.23) and PO_2_ (UB.1)

**Figure 11b:**
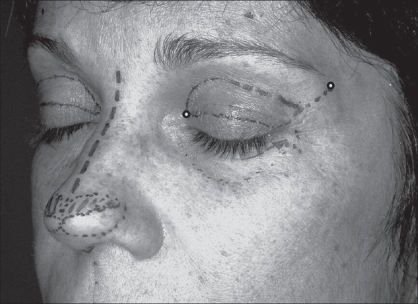
Surgical demarcation - excessive skin area of the upper eye lid to be trimmed was demarcated. The line hatched between the PO_1_ locus and lateral canthus commissure controls the lateral extension of the eyelid scar. It is also used to guide to the periostal fixation site of the lateral canthus tendon in canthopexy.

**Figure 11c:**
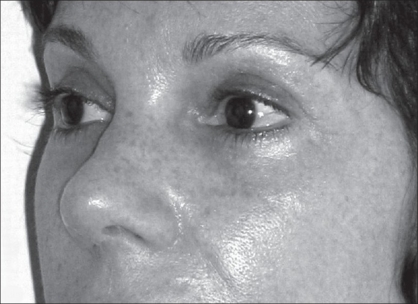
After one year follow-up, the upper eyelid scar of the patient was maintained inside the lateral orbital perimeter, allowing its concealment within the supratarsal fold.

## DISCUSSION

Beauty is a concept that diverges from cultural differences imposed by genetics, religion, geography, social influences or historical custom. According to Pitanguy,[36] form and structure, in a universal context, is rigid and orthodox; however, differences in race and cultures and the plurality of geographical locations have resulted in diverse aesthetic concepts. Each race possesses its own ideals of beauty. Furthermore, amongst people of the same race, each human being has his own individual concept of beauty, which is moulded by his temperament, culture, and sensibility.

Plastic surgery (PS) adopts a standard of beauty derived from Renaissance concepts as the foundation of perfection, through “readjustments” of the aesthetic units. Surgical planning is the fundamental step for any kind of operative procedure to be carried out. However, conventional demarcation in PS as a decisive factor in the goal of operative success is still subjective and suffers from a lack of anatomical reference. Therefore, the surgeon's concept of beauty, based on findings of lack of balance, disproportionalities, and asymmetries according to his own experiences and feelings, is the main parameter that he applies in his clinical analysis of the patient. To avoid the unpredictable aesthetic complications, it is necessary to have a careful and meticulous surgical planning, since it may lead to irreversible sequelae.

*Reduction Mammoplasty (including Mastopexy):* The M_1_ locus was determined by the ALM of *Lingshu*. This locus plays an important role as a location site of the level of the NACc, since it is considered as a physiological level of the nipple in a young and nulipara woman. In other words, the anatomical position of the NACc of every woman should coincide to the level of M_1_ or lie below this locus. Therefore, M_1_ may be used to control the level of the NACc. In this study, there was no case of breast surgery with abnormal NACc lifting (the nipple level lying above M_1_). Patients with small sagging breasts who submitted to mastopexy had the “Point A”[22] and NACc levels coincided to M_1_ without any great changes in its position after one year follow-up, in spite of having the breast lower pole roundness formation due to gravity effect[27] [Figure 10a–c]. However, in patients with hypertrophic breasts who underwent the reduction mammoplasty, the “Point A” and NACc level were lower (1-2 cm) than M_1_. This procedure was used to avoid the up-riding appearance of the NACc provoked by breast lower pole roundness. The formation of the breast lower pole roundness is due to the progressive downward migration of lipoglandular tissue, and it tends to be more pronounced in patients with large breasts. This mechanism could lead to the increase of the breast's vertical scar length, and give a false diagnostic of abnormal NACc lifting.[27]

*Nipple-areola reconstruction:*Dufourmentel and Mouly[25] suggested that the placement of the NAC has been arbitrarily determined. Murphy *et al.*[26] described a new and sophisticated technique of NAC repositioning which is based on two consistent ratios and the average sternal notch-to-nipple measurement was 21 cm, as a standard distance. The nipple plane is located 0.33 times the distance from the sternal notch to the pubis, and the internipple distance is 0.23 times the chest circumference to define an accurate nipple placement. In the present study, a patient with atelia associated to Ectodermic Dysplasia was submitted to the NAC reconstruction in two stages (nipple placement with conchal cartilage graft followed by areola tattoo). The NACc was determined by the PM and ALM as described in *Lingshu,* whereas the site of the nipple was located at the upper border of the 5^th^ rib and 2/3 (4 *cun*) the distance between the midsternum line to the acromial-clavicular angle (6 *cun*), which corresponds to M_2_. After one year follow-up the new NAC preserved a natural look without any deviative appearance. We also observed that according to *Lingshu* the NACc position is quite simple to be located in man since its demarcation is directly related to M_2_, while in woman it depends on M_1_ to obtain the projection of the nipple placement.

*Augmentation mammoplasty*: The undermining area is based on the surgical demarcation through the lateral projections of the gland when the breast is pressed against the wall.[27] It is important to know the physiological location of the small and moderate size breasts can be determined by the upper landmark at the level of 2^nd^ rib (M_3_) and the lower landmark at the level of 6^th^ rib (M_4_). The area to be undermined is usually larger than the implant base; 1 to 2 cm below the inframammary sulcus (M_5_) and 1 to 1.5 cm from the medial and lateral aspects of the implant.[27] In our study, M_3_, M_4_, and M_5_ (located by the ALM) were used to demarcate the undermining area in patients who wanted to obtain small breast augmentation (195–215 cc). The surgical procedures were carried out normally, and after one year follow-up the breasts preserved a natural look without any complication related to breast contracture, asymmetry or abnormal dislocation.

*Abdominoplasty*: Sinder[28] described a personal technique of abdominoplasty which was based on a subjective demarcation of an hexagonal-shaped abdominal region delimitated by a superior line crossing the upper board of the umbilicus, and an inferior line, the transverse suprapubic abdominoplasty line that extends laterally to the iliac crests. A short perpendicular line (2 cm) is marked on the superior line 5-6 cm from both sides of the umbilicus which correspond to their opposite sites marked parallely on the inferior line. After a careful dissection and resection of the exceeded dermoadipose tissue, the upper and the lower margins are joined together with suture under the guidance of those crossing points. In this study, using the pars of loci Ab_1_-Ab_2_ or Ab_3_-Ab_4_ in narrow and large waist patients, respectively, could reinforce those short perpendicular lines described by Sinder. Moreover, these loci could provide an easy demarcation method to determine precisely the abdominal region to be excised. These loci were located by the PM. The final results were pleasant, and the abdominal scar was symmetric without tension.

*Neoomphaloplasty:* Thorek[30] pointed out, the umbilicus lies either on the midline at the level of the iliac crests, or it is placed from 1 to 2 cm above to transtubercular level (at the level iliac spines). Pitman,[31] suggested that the distance from the umbilicus to anterior vulvar commissure should be 18-21 cm. In young and healthy men and women, the waist which is the narrowest circumference of the torso, is usually 2.5 cm cephalad to the umbilicus. In this study, three patients submitted to abdominoplasty associated with neoomphaloplasty with good results. The umbilicus new site was determined at 5 *cun* above the suprapubic region, according to the PM measurement described in *Lingshu.* Considering that the distance between the sternocostal angle and suprapubic region is consisted of 13 *cun*, if the *cun* measure is uniformly distributed along that anatomical segment.

*Rhytidoplasty:* According to Pitanguy[29] the facial skin flap is, conventionally, advanced by traction from the “Point A” in a cephaloposterior direction (45-60°) between the tragus and the Darwin's tubercle. The superficial muscular aponeurotic system (SMAS), responsible for the facial movements, is also lifted up and out diagonally. After the flap dissection, it is plicated to increase the rejuvenation appearance. However, these maneuvers may not be done precisely, because there are some patients who lack in definition of Darwin's tubercle landmark or others who have asymmetric face. In this study, 10 patients underwent the cervicofacial rhytidoplasty with the traction direction of the facial flap from the “Point A” and the SMAS guided from F_1_ to F_2_ or from F1 to F_3_ with excellent results. The used aesthetic-loci were determined by the ALM. We also observed that the symmetry in facial expression lines, mimics, and skin tension were preserved with natural look without abnormal deviation.

*Blepharoplasty:* Under the view of Pitanguy,[29] particular care to be taken in the blepharoplasty is the previous marking of the nasal and temporal ends (orbital perimeter) which can determine the final result of the scar length. It is also important that the lateral extent of the excision area should never lie outside the orbital perimeter in moderate excess of upper eyelid cases. Conventionally, the design of excess skin of upper eyelid area to be resected is mostly related to its breadth. Moreover, it lacks in reference, in order to control the upper lid scar length. Therefore, a well demarcation design must be conceived, in order to obtain a natural appearance result. In this study, 21 patients underwent the conventional blepharoplasty. The upper eyelid scar length and symmetry could be controlled under the reference of those two loci (PO_1_ and PO_2_) [Figure 9, 11a–b]. These aesthetic-loci were positioned by the ALM. The final result of the upper eyelid scar lay inside the lateral orbital perimeter, and allowing its concealment within the supratarsal fold [Figure 11c].

*Lateral Canthopexy:* According to Flowers,[32] there is a periorbitary site which is 9 mm caudal to the center of the orbital rim prominence near the frontozigomatic suture, or choose a location where the preoperative supratarsal fold crosses the orbital rim to guide the periosteal fixation of the lateral canthal tendon in canthopexy. However, this measure may be difficult to be reached symmetrically. An alternative way to determine the periosteal fixation site of the lateral canthus tendon is using a traction direction from the lateral palpebral commissure to PO_1_ (TE.23) which can guide, as shown in Figure 7. In this study, 10 patients underwent the blepharoplasty associated with lateral canthopexy without any aesthetic or functional complications.

*Baldness Correction (Hair Implant):* Some authors[33,34] suggested that one of the most important step of the baldness correction is the demarcation of the hair frontline (HFL). Conventionally, the midpoint of HFL, known as “Point H”[34] can be determined by different methods.*(a)* It can be located at least two fingerbreadth above the highest median frontal crease (this approximate point depending on the breadth of the surgeon's fingers), *(b)* It can be determined by using the rule of three equivalent distances: chin to base of nose, base of nose to root of nose, root of nose to AH, *(c)* It can be demarcated between 7 and 10 cm above the glabelar level [Figure 8b]. In this study, three patients ranging in age between 35 and 70 years underwent hair implant with fair and good results. The S_1_ locus was demarcated at 3 *cun* above glabelar region, on the forehead, according to TCM [Figure 8a]. Moreover, S_1_ is not an acupoint, but it is only used to describe the aesthetic location of the center midpoint of the HFL, which is, usually, coincided with the “Point H”.

*Ear Reconstruction:* According to Pitanguy[29] the demarcation of the ear's framework is based on horizontal plane projections of the anatomical landmarks of the face to the temporoparietal region to determine the future auricle location, such as: *(a)* the lateral border of the eyebrow to the origin of the helix;*(b)* the columella base to the attachment of the ear lobe, and (c) the tragus lying halfway between these two planes. In this study, there were two patients who underwent the ear reconstruction using ALS to locate and design the ear's framework for partial or total ear reconstruction. The ear's demarcation could be reinforced by using the horizontal plane projections of the anatomical landmarks under the reference of aesthetic-loci, such as: *(a)* E_1_ to E_2_ corresponds to the ear's apex; *(b)* E_3_ to E_4_ the ear's helix root; *(c)* E_5_ to E_6_, the ear's lobe attachment; (d) E_7_ and E_8_ are respectively the 1/3 and 2/3 the distance between E_2_ and E_6_ lying on the mastoid process which corresponds to the posterior aspect of the ear, approximately one eye *cun*. In the first case, a 7-year-old female with unilateral grade III microtia submitted to total auricular reconstruction with rib cartilage graft in two stages. The patient had a fair result due to the partial absorption of the cartilage that altered the size and shape of the new ear. The second case was a 23-year-old male with absence of upper third of the ear following trauma who underwent partial ear reconstruction with conchal cartilage graft covered by a randomized mastoid skin flap in two stages. After one year follow-up, patient had good result since the shape of the ear upper pole was maintained in size and shape but its external characteristics were deformed by scars. In general terms, the dimensions of the ear of these cases were preserved within the ear's framework area guided by those aesthetic-loci.

In spite of having categories of surgical procedures based on a few cases (NAC reconstructions, ear reconstructions, and hair implant) that are statistically inconsistent, they play an important role in PS in providing new possibilities of using aesthetic-loci in different type of aesthetic surgery.

*The importance of Oriental Anthropometry in Plastic Surgery:* SMAP is considered to be an anthropometric science (OA) because it comprises of a system of well-defined compartments using its own methods of measurement mainly based on DM, PM, and ALM, and employs the *cun* as the basic unit of reference measurement for the whole body surface. Theoretically, a perfect anatomical body would consist of sections in which the *cun* measure is the same for every part of the body.[37] However, this rarely occurs and the measures vary from one section of a body region to another. For instance, the *cun* of the forearm can be different from the *cun* of the leg.[6]

In accordance to recent studies by Coyle and Aird,[16] these traditional methods do not produce accurate acupoints locations, firstly because the *cun* unit concept is not a uniform measure of whole body sections, and secondly because the standard *cun* measure defined by these reference measurements varies from one section to another. Consequently, the greater the length of a section, the more *cun* units are involved and the greater the amplification of inaccuracy.[16] The ALM[17] is the most reliable method in locating acupoints because it is based on body structures such as folds, creases, depressions that lie between bones, muscles, tendons, vessels, etc. However, it is only useful for acupoints situated near these structures. For those points which are far from anatomical landmarks, it is usual to apply traditional reference measurements (PM and DM).[15,16] The PM divides a distance between two landmarks or reference marks into equal sized sections, while the DM measures the distance from one landmark or reference mark to an acupuncture point.[15,16] Even so, the former deserves more credibility due to its precision, flexibility, and applicability since it takes into account the anatomical references of the each person size, gender, age, and race. Although the OA appears to be a subjective view, it is, however, a systematic and well-organized science that can be used in PS as a safety measure using ALS to guide the surgical planning. For instance, we suggest the surgeons (specially, those who are inexperienced in proportionality and symmetry in AU) to use this concept in surgical demarcation, the results should be better with more precision in body measurements than using conventional methods. Throughout this study, we have observed that ALS is helpful in clarifying the surgical planning overview and in reinforcing conventional surgical demarcation. Operations have therefore been carried out more easily and safely.

*The importance of Oriental Anthropometry in Traditional Chinese Medicine:* In TCM, the theory of the meridians and energy points allied to the other fundamental concepts of TCM, such as energy balance of Yin-Yang; relationship between the organs and viscera - Zang-Fu; vital energy - Qi, blood and fluids; and (iv) Five Elements; have always been applied strictly in specialized therapeutic applications such as acupuncture, Tuina (Chinese massage), Oriental Herbology, Qigong (meditation and breathing exercises).[6]

The Oriental Anthropometry, as mentioned before, is a useful technique for anatomical guidance in PS because it is a compartmentalized system of proportionality and symmetry. However, there is no other previous study which deals with the use of SMAP as an anthropometric method, or vice-versa. Therefore, OA is also considered to be a new concept in TCM, because in its philosophy it differs from other innovations in acupuncture by using acupoints as aesthetic loci in PS for the purposes of anatomical reference, instead of applying them for therapeutic purposes.

## CONCLUSION

This work does not intend to prescribe which type of surgical marking is more suitable for the most common aesthetic procedures in Plastic Surgery (PS). However, the goal of this work is to introduce a new concept of body measurement (Oriental Anthropometry) as an anthropometric science, and a surgical demarcation method (Aesthetic Locus System) based on the proportionality and symmetry, which may provide patients with more reliable balance and harmony in facial and body contours. Therefore, it is suggested to be a useful tool for PS procedures. However, further rigorous randomized control trials to examine its effectiveness need to be performed.
